# Successful catheter ablation of postoperative atrial tachycardia with conduction disturbances: Assessment by late‐gadolinium enhancement magnetic resonance imaging and high‐resolution electro‐anatomical mapping

**DOI:** 10.1002/ccr3.4198

**Published:** 2021-06-17

**Authors:** Kazutaka Nakasone, Kunihiko Kiuchi, Mitsuru Takami, Yu Izawa, Koji Fukuzawa, Ken‐ichi Hirata

**Affiliations:** ^1^ Section of Arrhythmia Division of Cardiovascular Medicine Department of Internal Medicine Kobe University Graduate School of Medicine Hyogo Japan

**Keywords:** catheter ablation, late‐gadolinium enhancement magnetic resonance imaging, postoperative atrial tachycardia

## Abstract

Atrial tachycardia (AT) in the right atrium often occurs following open‐heart surgery. Catheter ablation for these AT is challenging and can lead to unintended conduction block. We performed late‐gadolinium enhancement magnetic resonance imaging (LGE‐MRI) prior to catheter ablation and predicted wavefront propagation during SR as well as the slow conduction zone during tachycardia. LGE‐MRI may assist predicting the conduction disturbance and reducing the risk of unexpected sinus exit block.

## Graphical abstract

Late‐gadolinium enhancement magnetic resonance imaging (LGE‐MRI) prior to catheter ablation of postoperative AT in RA can predict wavefront propagation during sinus rhythm as well as the slow conduction zone during tachycardia. It could reduce the risk of unexpected sinus exit block and PM implantation.

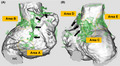

## 1 INTRODUCTION

Atrial tachycardia (AT) in the right atrium (RA) often occurs following open‐heart surgery and is difficult to treat. Although 3D mapping systems have improved treatment rates of AT, catheter ablation (CA) can lead to unintended conduction block.^1^ Early detection of fibrosis in RA is hence important. Recently, late‐gadolinium enhancement magnetic resonance imaging (LGE‐MRI) has been proposed as a useful tool for visualization of left atrial fibrosis.^2^ In this report, we describe a successful ablation of postoperative AT in the RA where LGE‐MRI accurately predicted the conduction disturbance.

## 2 CASE REPORT

A 53‐year‐old male was referred to our hospital with dyspnea, and 12‐lead electrocardiograms indicated AT. He had undergone surgery at the age of 9 years for tetralogy of Fallot. At the age of 49 years, he underwent tricuspid and mitral valvuloplasty, as well as pulmonary artery replacement for severe tricuspid, mitral, and pulmonary valve regurgitation, using a superior transseptal approach. On this occasion, the patient was diagnosed with tachycardia‐induced cardiomyopathy because his heart function dramatically improved after electrical cardioversion of the AT.

**FIGURE 1:**
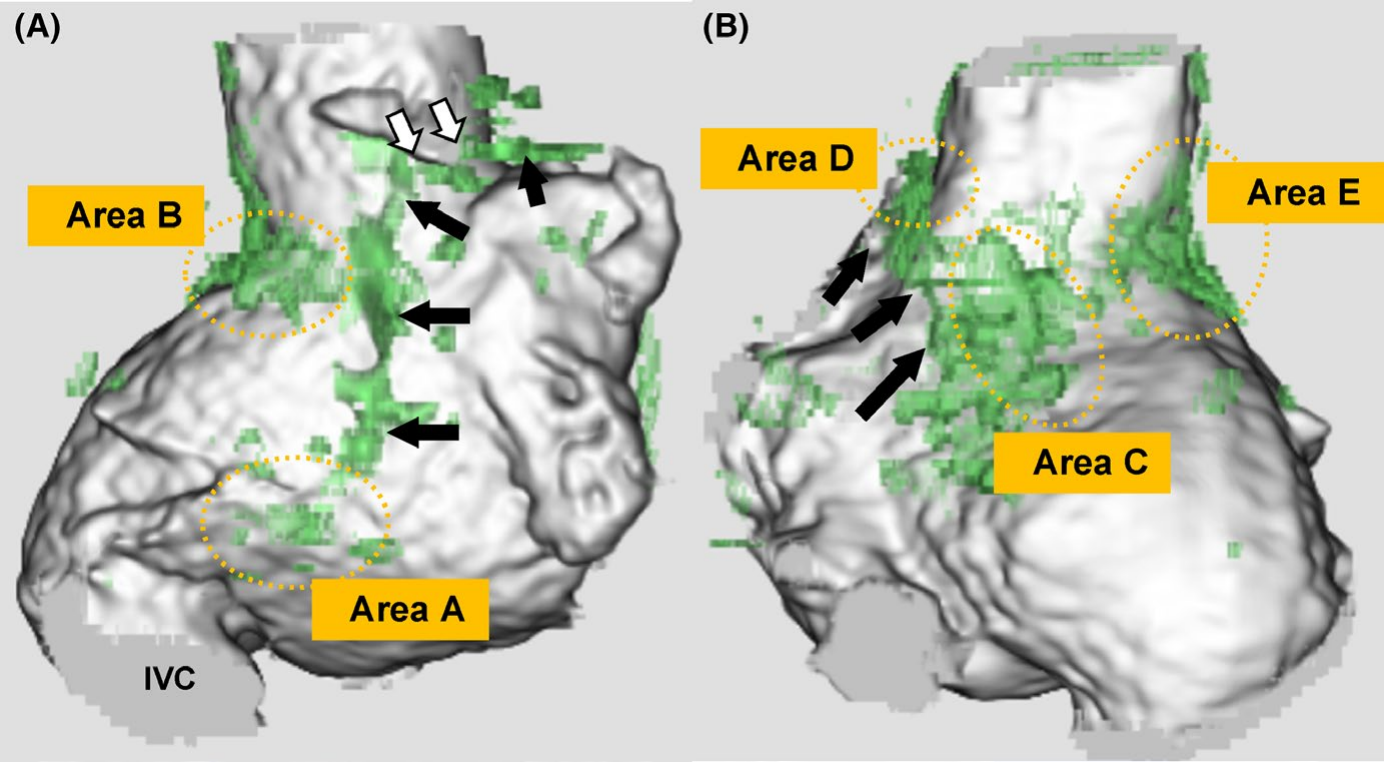
3D LGE‐MRI. 3D LGE‐MRI of the RA in the right anterior oblique (RAO) (A) and posterior‐anterior (PA) (B) views. The green areas indicate LGE. The black arrows show the long linear LGE, and the yellow dotted circles show the patchy one. The white arrows indicate the visual gap of the LGE. LGE‐MRI, late‐gadolinium enhancement magnetic resonance imaging; RA, right atrial; RAO, right anterior oblique; PA, posterior‐anterior

**FIGURE 2:**
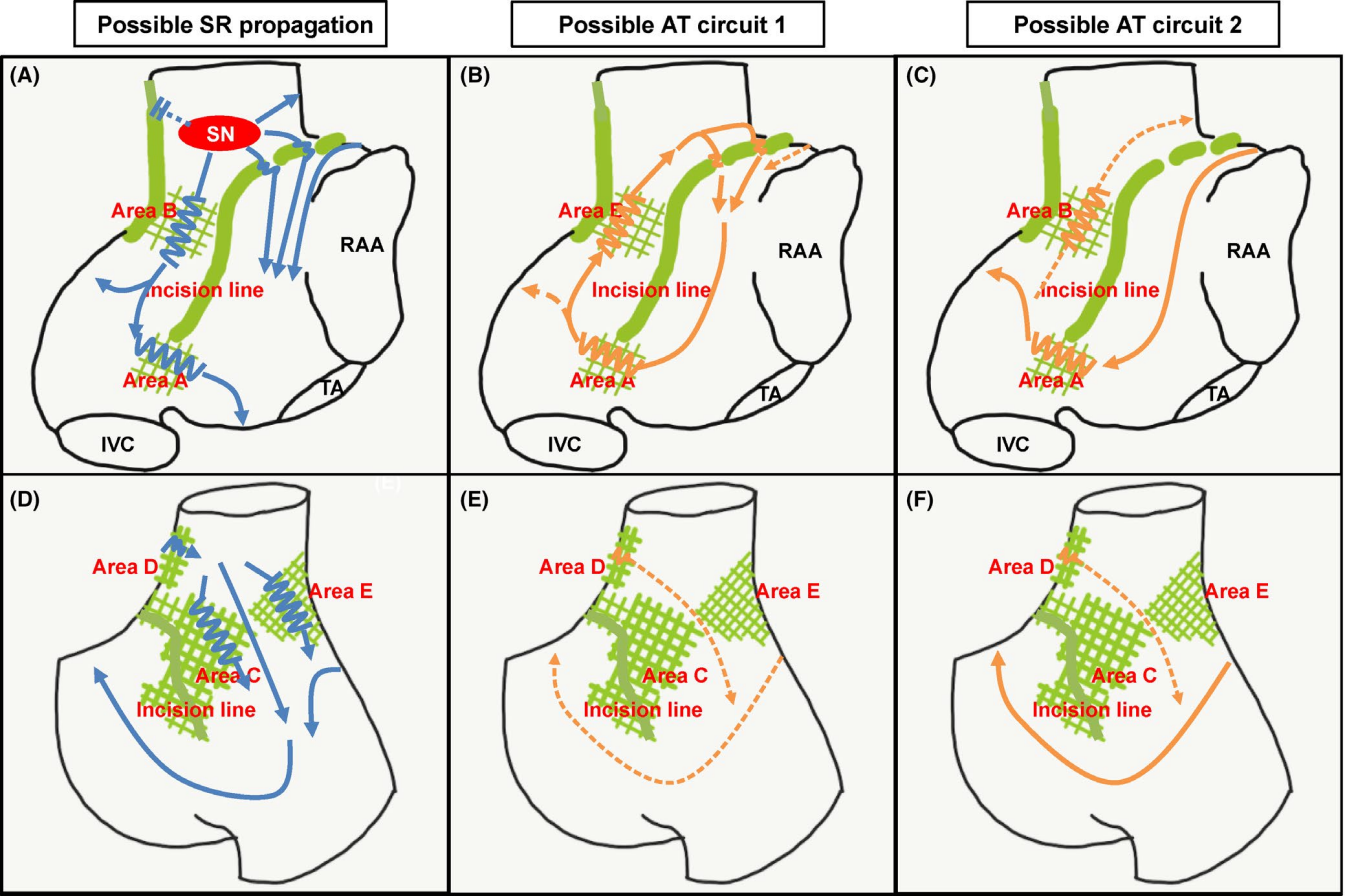
The possible propagation during SR and AT. The left panel shows the possible SR propagation in the RAO (A) and PA (D) views. The wavefront propagation from the SN was assumed in three directions. The first one was to propagate to the upper edge of the incision line, the second one was to propagate downward from the SN and bifurcated posteriorly and laterally, and the last one was to propagate through the gap of the incision line. The middle one shows one of the possible AT circuits in RAO (B) and PA (E). The wavefront propagates the RA free wall around the incision line through the gap. The right one shows one more possible AT circuit in RAO (C) and PA (F). The wavefront turns from the free wall to the septum around the linear LGE. LGE‐MRI, late‐gadolinium enhancement magnetic resonance imaging; SR, sinus rhythm; AT, atrial tachycardia; RA, right atrial; SN, sinus node; RAA, right atrial appendage; TA, tricuspid annulus; IVC, inferior vena cava; RAO, right anterior oblique; PA, posterior‐anterior

To prevent AT recurrence, CA was conducted. LGE‐MRI was performed prior to CA. For better visibility, LGE‐MRI was three‐dimensionally reconstructed and fused with the contrast enhancement MR angiography (CE‐MRA) as described previously.^3^ LGE was detected by using atrial wall‐based thresholding methods of above 1SD. LGE‐MRI showed linear or patchy late‐gadolinium enhancements (LGEs) in the RA (Figure 1A and B). The long linear LGE reached from the RA free wall to the septum, and an LGE gap was partly visible near the sinus node (SN; Figure 1A and B, black arrows: long linear LGE, and white arrows: visual LGE gap). Furthermore, five patchy LGEs were found around the linear LGE: between the linear LGE and the inferior vena cava (Area A), below the SN (Area B), and around the posterior septum (Areas C, D, and E) (Figure 1A and B). Based on the LGE‐MRI, we speculated the wavefront propagation during sinus rhythm (SR) and tachycardia circuits (Figure 2). The SN was surrounded by the long linear LGE and patchy LGEs (Areas B and D), where radio frequency (RF) application potentially risked isolation of the SN (Figure 2A and D). On the other hand, the atrioventricular node (AVN) was activated by two pathways through the low lateral RA (Area A) and the posterior RA (Area C, E). Therefore, we considered that RF application at the low lateral RA (Area A) could be performed without compromising safety.

Concerning the possible AT circuit, in addition to cavotricuspid isthmus‐dependent atrial flutter, the following two macro‐re‐entrant ATs associated with the long linear LGE were considered: (a) incisional AT1, which turns around at the visual LGE gaps (Figure 2B and E) and (b) incisional AT2, which turns around at the septum edge of the long linear LGE (Figure 2C and F). If the conduction velocity was decreased in Area A, it was considered a possible ablation target, as both incisional AT1 and 2.

**FIGURE 3:**
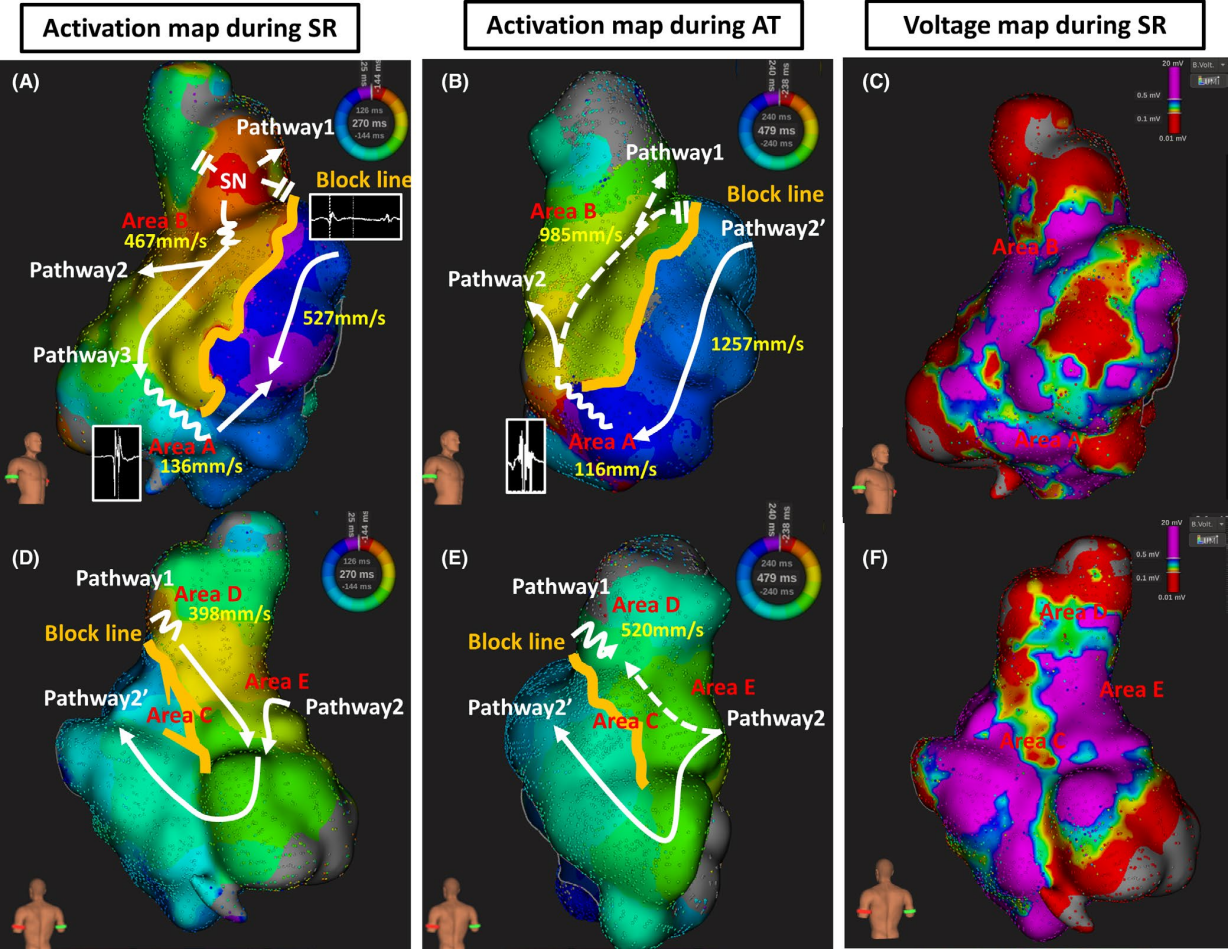
Activation map during SR and AT, and voltage map during SR. The left panels show the activation map during SR of the RA in the RAO (A) and PA (D) views. The total activation window was divided into 15 isochronal zones, from red to purple. The wavefront propagation extended from the SN in three directions. Pathway 1 propagates superiorly with conduction delay at the superior edge of the incision line. Pathway 2 also propagates posteriorly from the SN. Subsequently, Pathway 1 and 2 collided at the RA posterior wall and propagated to the anteroseptal wall after the turn at the septal end of the linear LGE. Pathway 3 propagates caudally along the linear LGE and slowly turns around the edge of the linear LGE. Pathways 2 and 3 collided with the low lateral RA. The conduction velocities at each site during SR were 136 mm/s in Area A, 467 mm/s in Area B, 398 mm/s in Area D, and 527 mm/s at the site without LGE. The middle panels show the activation map during AT of the RA in the RAO (B) and PA (E) views. The activation map demonstrated that the activation propagated clockwise around the long linear LGE. The total activation window was also divided into 15 isochronal zones. Pathway 1 showed slow conduction at the upper edge of the incision line during AT, conducted superiorly, and then collided with Pathway 2. Pathway 2 was the main circuit during AT, which was conducted posteriorly through the sinus venosa. Subsequently, it propagated to the anteroseptal wall after the turn at the septal end of the LGE. The conduction velocities at each site during AT were 116 mm/s in Area A, 985 mm/s in Area B, and 520 mm/s in Area D, and 1257 mm/s at the site without LGE. The right panels show the voltage map during SR of the RA in the RAO (C) and PA (F) views. The red and purple indicated that atrial voltage of 0.1 and 0.5 mV, respectively. The atrial voltage between 0.1 and 0.5 mV was colored. SR, sinus rhythm; AT, atrial tachycardia; RA, right atrial; RAO, right anterior oblique; PA, posterior‐anterior; SN, sinus node; LGE, late‐gadolinium enhancement

An automated high‐resolution mapping system (Rhythmia, Boston Scientific) clearly demonstrated wide double potentials at the linear LGE reaching from the RA free wall to the septum during SR. This indicated conduction block along the linear LGE (Figure 3A and D). The activation from the SN propagated posteriorly or caudally behind the linear LGE. Subsequently, these two activations turned around both edges of the linear LGE and collided at the tricuspid valve site of the low lateral RA. Notably, a slightly fragmented atrial voltage was found at the patchy LGE (Area A), and its conduction velocity was extremely slow compared to the other patchy LGEs. Therefore, the AVN was mainly activated by a posteriorly propagated pathway rather than a caudally propagated pathway. The voltage map demonstrated that the patchy LGE regions corresponded to the low voltage areas where the atrial voltage was less than 0.5mV. Particularly, the atrial voltage at the linear LGE region was significantly low (<0.1 mV; Figure 3C and F).

Clinical AT (cycle length: 476 ms) was easily induced by burst pacing, and the rhythmic system demonstrated that the activation propagated clockwise around the long linear LGE (Figure 3B and E). The fractionated potentials could be recorded at the patchy LGE (Area A), where the single RF application could terminate the AT without prolongation of the PR interval. Subsequently the cavotricuspid isthmus ablation was added, because the cavotricuspid isthmus‐dependent atrial flutter was also induced. At the end of the procedure, no AT was induced. The patient has been free from AT recurrence for eight months after CA.

## 3 DISCUSSION

Our case report demonstrated the usefulness of LGE‐MRI to assess conduction delay or block during SR and tachycardia in postoperative patients. To the best of our knowledge, there has been no previous report of the relationship between LGE‐MRI and conduction disturbances in postoperative RA.

AT in the RA after open‐heart surgery is difficult to diagnose and treat because of the effects of scar tissue associated with surgery and pre‐existing functional conduction block specific to the RA. In addition, CA has a potential risk of unintended localized conduction block in the RA. Furthermore, in some cases, isolation of the lateral RA may result in complete sinus exit block requiring pacemaker implantation.^1^ Although this might be prevented if wavefront propagation during SR was assessed in advance, in many cases the RF application is performed during tachycardia. Therefore, it is beneficial to assess fibrosis distribution of the RA prior to the procedure.

Recently, LGE‐MRI has been proposed as a method to assess atrial fibrosis.^2^ Cardiac fibrosis leads to conduction delay and local conduction block, which promote functional re‐entry.^4^ It has been reported that increased left atrial LGE is associated with lower local conduction velocity.^5^ In the our case, LGE‐MRI was performed in the RA. This showed a correlation between the LGE sites and conduction delay or block in high‐resolution mapping. Comparing the LGE‐MRI and electro‐anatomical mapping, the conduction velocities were slower in Areas A and D than in Area B, despite similar patchy LGE sites. This discrepancy may be caused by the direction of wavefront propagation. Animal studies have shown that longitudinal conduction velocities are 2‐10 times faster than transversal velocities in the RA, owing to a uniform longitudinal arrangement of muscle fibers and non‐uniform distribution of connexins to the interstitial collagen.^6^ This suggests that, although fibrosis causes conduction delay, transversal fibrosis causes a more pronounced conduction delay and may form the basis of the re‐entry circuit.

Linear LGE appeared to have a visual gap in the superior right appendage on LGE‐MRI; however, no conduction across the line during both SR and AT could be detected. A previous report showed that the visual gap length in the PV ablation line on LGE‐MRI was associated with AF recurrence after PV isolation, and the threshold for the highest specificity was a gap length of 4.3 mm.^7^ In our case, the gap length on LGE‐MRI was 2.7 mm. Therefore, we should have speculated that these small visual gaps might demonstrate a conduction block.

## 4 CONCLUSIONS

By using the LGE‐MRI prior to CA, wavefront propagation during SR as well as the slow conduction zone during tachycardia could be assessed. After CA of postoperative AT, this may assist in reducing the risk of unexpected sinus exit block and it may negate the need for pacemaker implantation.

## CONFLICT OF INTEREST

All authors declare no conflicts of interest for this article.

## ETHICS STATEMENT

The study was approved by the local ethics committee and complied with the Declaration of Helsinki (Committee of 2014.10.31., Approval No. 1663).

## Data Availability

The data underlying this article will be shared upon reasonable request to the corresponding author upon approval from the Ethics Committee of the institution.
